# Frontiers in Anti-Cancer Drug Discovery: Challenges and Perspectives of Metformin as Anti-Angiogenic Add-On Therapy in Glioblastoma

**DOI:** 10.3390/cancers14010112

**Published:** 2021-12-27

**Authors:** Laura Guarnaccia, Giovanni Marfia, Matteo Maria Masseroli, Stefania Elena Navone, Melissa Balsamo, Manuela Caroli, Silvia Valtorta, Rosa Maria Moresco, Rolando Campanella, Emanuele Garzia, Laura Riboni, Marco Locatelli

**Affiliations:** 1 Laboratory of Experimental Neurosurgery and Cell Therapy, Neurosurgery Unit, Foundation IRCCS Ca’ Granda Ospedale Maggiore Policlinico, 20122 Milan, Italy; laura.guarnaccia@policlinico.mi.it (L.G.); matteo.masseroli@unimi.it (M.M.M.); stefania.navone@policlinico.mi.it (S.E.N.); melissa.balsamo@policlinico.mi.it (M.B.); manuela.caroli@policlinico.mi.it (M.C.); campanella.rolando@gmail.com (R.C.); marco.locatelli@policlinico.mi.it (M.L.); 2 Department of Clinical Sciences and Community Health, University of Milan, 20122 Milan, Italy; 3 Clinical Pathology Unit, Aerospace Medicine Institute “A. Mosso”, Italian Air Force, 20138 Milan, Italy; 4 Department of Medicine and Surgery and Tecnomed Foundation, University of Milan Bicocca, 20900 Milano, Italy; silvia.valtorta@unimib.it (S.V.); Moresco.rosamaria@hsr.it (R.M.M.); 5 Department of Nuclear Medicine, San Raffaele Scientific Institute, IRCCS, 20132 Milano, Italy; 6 Institute of Bioimaging and Molecular Physiology, National Research Council (IBFM-CNR), 20090 Segrate, Italy; 7 Aerospace Medicine Institute “A. Mosso”, Italian Air Force, 20138 Milan, Italy; Emanuele.garzia@aeronautica.difesa.it; 8 Reproductive Medicine Unit, Department of Mother and Child, San Paolo Hospital Medical School, ASST Santi Paolo e Carlo, 20142 Milan, Italy; 9 Department of Medical Biotechnology and Translational Medicine, Laboratorio Interdisciplinare Tecnologie Avanzate (LITA)-Segrate, University of Milan, 20054 Milan, Italy; laura.riboni@unimi.it; 10 Department of Medical-Surgical Physiopathology and Transplantation, University of Milan, 20122 Milan, Italy; 11 “Aldo Ravelli” Research Center, 20142 Milan, Italy

**Keywords:** brain tumors, glioblastoma, angiogenesis, metformin

## Abstract

**Simple Summary:**

Glioblastoma is the most aggressive primary brain tumor, with the highest incidence and the worst prognosis. Life expectancy from diagnosis remains dismal, at around 15 months, despite surgical resection and treatment with radiotherapy and chemotherapy. Given the aggressiveness of the tumor and the inefficiency of the treatments adopted to date, the scientific research investigates innovative therapeutic approaches. Importantly, angiogenesis represents one of the main features of glioblastoma, becoming in the last few years a major candidate for target therapy. Metformin, a well-established therapy for type 2 diabetes, offered excellent results in preventing and fighting tumor progression, particularly against angiogenic mechanisms. Therefore, the purpose of this review is to summarize and discuss experimental evidence of metformin anti-cancer efficacy, with the aim of proposing this totally safe and tolerable drug as add-on therapy against glioblastoma.

**Abstract:**

Glioblastoma is the most common primitive tumor in adult central nervous system (CNS), classified as grade IV according to WHO 2016 classification. Glioblastoma shows a poor prognosis with an average survival of approximately 15 months, representing an extreme therapeutic challenge. One of its distinctive and aggressive features is aberrant angiogenesis, which drives tumor neovascularization, representing a promising candidate for molecular target therapy. Although several pre-clinical studies and clinical trials have shown promising results, anti-angiogenic drugs have not led to a significant improvement in overall survival (OS), suggesting the necessity of identifying novel therapeutic strategies. Metformin, an anti-hyperglycemic drug of the Biguanides family, used as first line treatment in Type 2 Diabetes Mellitus (T2DM), has demonstrated in vitro and in vivo antitumoral efficacy in many different tumors, including glioblastoma. From this evidence, a process of repurposing of the drug has begun, leading to the demonstration of inhibition of various oncopromoter mechanisms and, consequently, to the identification of the molecular pathways involved. Here, we review and discuss metformin’s potential antitumoral effects on glioblastoma, inspecting if it could properly act as an anti-angiogenic compound to be considered as a safely add-on therapy in the treatment and management of glioblastoma patients.

## 1. Introduction

Central nervous system (CNS) tumors are a group of different neoplastic entities, that although arising in the same anatomical location, are very heterogeneous for morphology, etiology, site, molecular biology and clinical behavior [1]. They are frequently characterized by high morbidity and mortality, depending also on their localization, grade and rate of invasive growth [2]. Most neoplastic brain lesions, known as secondary tumors, are metastases arising from cancers outside the CNS, being 5–10 times more frequent than primary brain tumors [3]. Among the primary brain tumors which arise without previous lesions, gliomas and meningiomas are the most common types [4]. Gliomas are primitive CNS tumors so called for their origin from glial cells or glial cell precursors [5]. Among all gliomas, surely the most malignant and frequent lesion is glioblastoma (WHO grade IV), which alone represents around the 45–50% of all the malignant primary tumors of the CNS. Its incidence rate is 3/100,000 cases per year, but it increases with age (reaching a peak of 15/100,000 cases per year in people aged 75–84 years old), male gender and white Caucasian race [6]. The median survival rate of patients affected by a newly diagnosed glioblastoma is around 14.6 months, mainly because the gold standard therapy has a low impact on its mortality and on the progression free survival (PFS); recurrence is, therefore, the rule and the outcome is invariably fatal [7].

Based on the molecular features, Verhaak et al. have described four different phenotypes; Classical, Neural, Proneural and Mesenchymal. The classical subtype shows aberrant alterations, including amplification of Chr7, loss of Chr10, inactivation of the RB (Retinoblastoma-associated protein) pathway and focal 9p21.3 homozygous deletion. In addition, Sonic hedgehog pathways, Notch signaling pathways and the neural precursor and stem cell marker NES are highly expressed in the classical subtype. Importantly, patients with the classical subtype show a significant reduction in mortality with aggressive radiotherapy and chemotherapy. The neural subtype has similar gene expression patterns compared with normal brain tissue and shows neural markers as NEFL (Neurofilament light polypeptide), SLC12A5 (Solute carrier family 12 members 5), SYT1 (Synaptotagmin 1) and GABRA1 (Gamma-aminobutyric acid type A receptor alpha1). Notably, it often tends to be more responsive to radiation and chemotherapy. The proneural subtype is characterized by high PDGFRA gene expression and frequent IDH1 mutation and is found primarily in younger patients. Despite showing no significant difference from other subtypes in response to chemotherapy and radiotherapy, the proneural subtype has the better survival rate [8]. The mesenchymal subtype is characterized by extensive necrosis and inflammation, upregulation of interstitial and angiogenesis genes, deletion of tumor suppressor genes P53, PTEN and NF1, and high expression of genes in the tumor necrosis factor superfamily and the NF-κB pathway [9]. Although responsive to aggressive radiotherapy and chemotherapy, the prognosis of mesenchymal subtypes is the worst among all subtypes [8,9,10]. The 2016 WHO classification for gliomas introduced molecular profiling, in addition to the traditional histopathological definition, evaluating markers with predictive and/or prognostic value, as the methylation status of O6-methylguanine (O6-MeG)-DNA methyltransferase (MGMT) promoter, mutational status of IDH1/2, and presence of 1q-19q codeletion [4]. Several clinical and molecular are currently considered as favorable prognostic indicators, for example an age at diagnosis < 50 years, MGMT methylation (>9%), Karnofsky Performance Status (KPS) > 70, gross/subtotal resection (>90%) and the tumor being in a non-eloquent area of the brain [11]. Notably, recent studies revealed that several low-grade *IDH*-wt gliomas with molecular features of glioblastoma are now up-classified to “glioblastoma”, for their aggressive clinical behavior, leading to their reclassification in 2021 WHO guidelines [12].

One of the most common hallmarks responsible for glioblastoma malignancy is angiogenesis, a mechanism that allows tumor mass vascularization and infiltration into surrounding tissues, thanks to the formation of novel and disorganized blood vessels which provide oxygen and nutrients to sustain tumor growth. For its large contribution to glioblastoma morbidity, angiogenesis has rapidly become a target of molecular target therapy. In 2009, Bevacizumab (Avastin^®^, Genentech/Roche), a monoclonal antibody against human vascular endothelial growth factor (VEGF), the most characterized proangiogenic factor, entered clinical practice thanks to quick US Food and Drug Administration (FDA) approval, as a monotherapy for the treatment of patients with recurrent glioblastoma. Unfortunately, the initial promising radiographic response, the increased response rates and the six-months increase of PFS have not been subsequently confirmed, so most patients treated with bevacizumab experienced a recurrence in three to five months [13,14]. Inevitably, the need to develop an effective treatment approach to fight glioblastoma animated a great number of studies to deepen the knowledge on the pathogenesis of glioblastoma and the underlying cellular and molecular mechanisms potentially targetable. Here, we focused on the observation of significant preventive and beneficial anti-cancer effect of metformin, which suggested the possibility to use metformin as an add-on therapy in many cancer subtypes, including glioblastoma [15]. Several studies had already shown a worsening of the prognosis and a decrease in survival in patients with glioblastoma and hyperglycemia, whether it was linked to a pre-existing diabetes mellitus, or whether it was a meta-steroid diabetes linked to therapy with corticosteroids [16]. Metformin (N, N-dimethybiguanide) is the most used anti-hyperglycemic drug all over the world, being the current first line therapy for all patients with newly diagnosed Type 2 Diabetes (T2D) [17]. Similar to phenformin and butformin (both withdrawn from the market since early 70s), it belongs to the biguanides class (molecules containing two linked guanidine rings), synthesized for the first time in 1922. However, due to the contemporary in-lab synthesis of insulin, which was considered the greatest development in the therapy of Diabetes Mellitus (DM), the drug did not get much consideration [18]. It was only after Jean Sterne’s studies in the mid-50s that metformin started gaining the attention it deserved: it was very helpful in treating patients with diabetes diagnosed in adult age, while it was inferior to insulin in treatment of diabetes of young patients [19]. This drug has found favor among clinicians because of its safety profile, availability, low cost, simplicity of administration and positive effects on body weight [20]. It was only a matter of time, therefore, that metformin was approved all over the world, starting in the UK (1958), then Canada (1972) and, finally, the USA (1998); after the UKPDS (United Kingdom Prospective Diabetes Study), which demonstrated an improvement in morbidity and mortality in diabetic patients treated with metformin, in 2009 this drug has been recommended as a first line therapy in the treatment of T2D by both the ADA (American Diabetes Association) and the EASD (European Association for the Study of Diabetes) [21,22]. Lately, some observations have led to the hypothesis that metformin could be repurposed because of its antineoplastic activity in vitro, shown in many tumors, including lung, pancreatic, colorectal, prostate and breast cancer and glioblastoma, giving rise to a new era of studies managing to deepen the knowledge of its effect [23,24]. The purpose of this review is to collect and discuss the scientific literature about metformin’s antitumoral effects in glioblastoma, while also suggesting that it could properly act on neo-angiogenesis, as proven in other tumors.

## 2. Neo-Angiogenesis in Glioblastoma

Angiogenesis is the highly sensitive and complex mechanism, by which the tumor mass sustains its progression with the formation of disorganized and unstructured blood vessels providing oxygen and nutrients. Tumor angiogenesis plays a key role in many physiological and pathological mechanisms and results from the interaction between different signaling pathways: it rapidly starts as a consequence of a hypoxic or ischemic condition, developing through the interaction between endothelial (ECs) and non-endothelial cells and components of extracellular matrix (ECM) [25]. In detail, this process requires the paracrine and autocrine activity of some soluble factors produced by the cells themselves, which determines the morphological modification of the ECs and the degradation of the ECM [26]. In cancer pathogenesis, particularly in high grade tumors (such as glioblastoma), aberrant neo-angiogenesis is a vital process for the mass growth: it is driven by neoplastic cells in order to respond to the tumoral hypoxic environment, which increases the demand for oxygen and nutrients by neoplastic cells, and is, therefore, essential to carry out the metabolic functions on which their survival is based [27]. On the other hand, several observations led to the knowledge that tumoral neo-angiogenesis gives rise to ultra-structurally abnormal vessels: most of them are dilated, convoluted and exceptionally permeable due to the presence of fenestrations and the lack of a complete basal membrane. Moreover, it is common that the vessel walls consist of a mosaic of ECs and cancer cells. The structural anomalies reflect the pathological induction and the tumoral ability of using common physiological mechanisms with the aim of boosting the mass growth.

### 2.1. Cell Biology of Glioblastoma Angiogenesis

**Figure 1:**
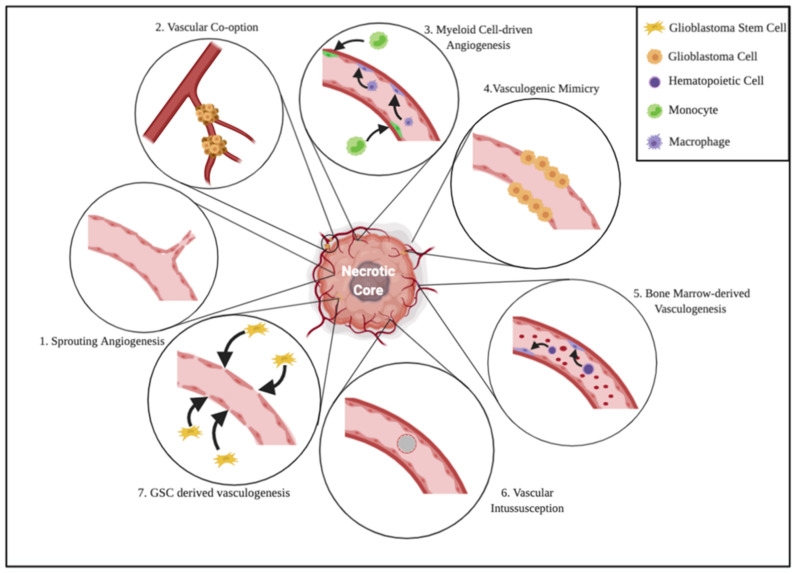
Cell biology of glioblastoma angiogenesis. As previously stated in the text, the relevance of some of these mechanisms in this kind of tumor remains uncertain. 1. Sprouting Angiogenesis: the mechanism by which capillaries undergo sprouting from pre-existing vessels. 2. Vascular Co-option: the process of infiltration of tumor cells into normal tissue exploiting pre-existing vasculature. 3. Myeloid Cell-driven Angiogenesis: M2 polarized monocytes/macrophages, which are able to polarize into EC phenotype. 4. Vasculogenic Mimicry: tumor cells replace ECs and form a vessel with a lumen. 5. Bone Marrow-derived angiogenesis: the enrollment of circulating progenitor endothelial cells to the tumor mass and the integration into vessel wall by transdifferentiation into mature ECs. 6. Vascular Intussusception: the formation of new vessels by vascular invagination, intraluminal pillar formation and splitting. 7. GSC-derived Vasculogenesis: Glioblastoma stem-like cells that contribute to the vascular neoformation by integrating into the walls and transdifferentiating into ECs.

In 2000, a study by Jain et al. reported six cellular mechanisms involved in tumor angiogenesis: (i) classical sprouting, (ii) vessel intussusception, (iii) vascular co-option, (iv) vasculogenic mimicry, (v) cancer stem-like derived vasculogenesis and (vi) bone marrow derived vasculogenesis [28]. More recently, the existence of a seventh mechanism has been demonstrated in the process of angiogenesis driven by blood derived infiltrating myeloid cells (Figure 1). Whether and how all the above-mentioned mechanisms are involved in gliomas or glioblastoma angiogenesis is not yet clear. What has been proven is that classical sprouting angiogenesis (the sprouting of capillaries from pre-existing vessels, known to be the most important mechanism in brain vascularization), vascular co-option (the mechanism of infiltration of tumor cells into surrounding normal tissue through pre-existing vasculature) and vasculogenic mimicry (the mechanism by which tumor cells form a lumen vessel by replacing normal ECs) are strictly involved in glioblastoma angiogenesis, giving the tumor its characteristic invasiveness [29]. On the other hand, experimental studies in glioma models have led to a conclusion that the importance of bone marrow-derived and cancer stem-like cell derived vasculogenesis, the mechanisms by which circulating progenitor endothelial cells and cancer stem-like cells get integrated into vessel wall by transdifferentiating into ECs, needs to be better clarified, as it appears highly controversial. Similarly, vessel intussusception by which the new vessels are generated by vascular invagination and splitting, and angiogenesis driven by bone marrow derived cells, as M2 polarized monocytes/macrophage appear to be rare events in tumor angiogenesis, needing deeper investigation [29]. However, it is well known that glioblastoma stem cells (GSCs) and glioblastoma endothelial cells (GECs) share a symbiotic and bidirectional relationship to maintain both angiogenic process and cell stemness. In particular, the glioblastoma hypoxic microenvironment induces the expression of hypoxia-inducible factor (HIF) in both cell subpopulation, generating a downstream cascade of events that promotes the synthesis and the paracrine release of some factors, as vascular endothelial growth factor (VEGF) and angiopoietins, by GSCs towards GECs, allowing cell proliferation [30,31]. Mainly through this mechanism, GSCs have the ability of remodeling the perivascular niche, by actively joining the formation of new vessels and/or by getting involved in maintaining GECs phenotype [32]. On the other hand, GECs also play an active role in maintaining GSCs stemness by acting on the downstream pathway Notch (which has a vital involvement in maintaining cell stemness) through the expression of delta like ligand 4 (DLL4) or Jagged1, both inducing a sustained activity of the receptor [33,34,35]. Moreover, it has been proven that GECs have also the ability of producing nitric oxide (NO) through the vascular synthase eNOS/NOS3; this molecule plays a role in promoting Notch signaling, thus promoting the stem phenotype [36,37]. Therefore, it is understandable because of what has been mentioned that a good anti-angiogenic therapy cannot prescind from also having an effective action on GSCs.

### 2.2. Angiogenic Signaling Pathways in Glioblastoma

In glioblastoma, many signaling pathways activated by the bond between growth factors and their receptors have been thoroughly studied with the aim of identifying possible targets for antiangiogenic therapies, leading to a better knowledge of their mechanisms. Among them, VEGF is the main angiogenic factor in CNS, fundamental in both embryonic development and tumor growth. In mice, the deletion of one of its variants or even of one of its receptors (VEGFR) results in immediate embryonic death due to severe defects in vascular system development [38,39]. VEGFR2 is the main receptor mediating several physiological and pathological effects of VEGF, favoring survival and proliferation of GECs; during angiogenesis, vessels start dilating and become weaker because of the action of such growth factor produced by neoplastic cells. Angiopoietin, together with other minor proteinases, stimulates this process by dissolving the ECM, proportionally with the increased secretion of VEGF. Throughout the mechanism, the action of these two molecules is vital: it is well known that their presence allows the survival of quiescent GECs even for years, enabling the development of new vessels when favorable conditions arose [40]. Moreover, VEGF together with granulocyte macrophage-colony stimulating factor (GM-CSF), insulin-like growth factor (IGF1) and angiopoietins 1 and 2 are all implicated in the mobilization of endothelial precursor. Intra-tumoral levels of VEGF in gliomas and its receptor strongly correlates with the histological grade of the tumor. In glioblastoma, particularly in the pseudopalized necrotic region, VEGF is upregulated [40,41,42,43]; this condition is mainly driven by HIF family, which is overexpressed in the central necrotic core of the tumor because of its hypoxic microenvironment. VEGF-induced angiogenesis leads to dysfunctional and immature vessels production, associated with significant oedema and disruption of blood-brain barrier (BBB) [44]. The increased secretion of this factor, together with its relationship with HIF, is currently thoroughly studied because of their possible implications in antiangiogenic therapy: the rationale is that making them targets of the treatment could drive promising therapeutic responses and improve overall survival (OS) and PFS. In this scenario, however, it is useful to acknowledge that in vivo studies on murine models and clinical trials on the treatment with bevacizumab (a monoclonal antibody targeting VEGF) have led to the observations that some aggressive and resistant cellular clones (able to form pseudopods and to migrate) are selected by the therapy, giving rise to tumor relapse. Studying this phenomenon has led to the identification of c-met as the vital gene for these clones to survive; as a matter of fact, the gene is upregulated because of the hypoxic microenvironment that starts phosphotyrosine phosphatase (PTP1B) pathway as a response to the reduction of VEGFR activity mediated by bevacizumab. However, for this process to happen the co-expression of both PTP1B and c-met is vital [29]. Another important molecule involved in the various pathways leading to angiogenesis is Notch. This protein is well-known for being intercalated on different signaling pathways leading to organ development and, more recently, some of its receptors (particularly Notch 1 and (4) have been recognized on EC membrane. Notch, together with VEGF, is vital in determining differentiative pathways of the precursor of the ECs, which can become either a tip cell or a stalk cell. VEGF-A causes an increase in VEGFR2 and 3 signals, leading to the development of tip cells; consequently, these cells cause the overexpression of the adjacent of Notch receptors, leading to the differentiation into stalk cells because of the interaction with DLL4 [45]. This last molecule is present in glioblastoma but not in glioma cells, demonstrating once again the importance of the neo-angiogenic activity particularly in these grade IV tumors [46]. Finally, deepening the knowledge of how the pathway mediated by angiopoietin and its receptor Tie2 works has gained interest, particularly because of the discovery that, by modulating it, an alteration in the structure of the vessels and the inhibition of the tumor growth is obtained. The tyrosine-kinase linked with the Tie2 receptor is expressed in the ECs and in some hematopoietic cell subtypes during their development and is a critical protein in vascular development. Unlike VEGFR, which is mostly or totally downregulated in adults’ vascularization, Tie2 is normally expressed and phosphorylated, promoting vascular stabilization by pericytes. Angiopoietins, particularly 1 and 2, on the other hand bind Tie2 with opposite effects between them: angiopoietin 1 activates it, angiopoietin 2 inhibits it [29]. The activation results in vascular stabilization and permeability decrease, vital processes for vessels development in the sane patient. Moreover, it has been observed that angiopoietin 2, particularly over-expressed in glioblastoma which favors the formation of immature vessels at the beginning of the angiogenesis, has a pro-inflammatory activity that leads to the recruitment of myeloid cells; these are involved in neovascularization process and in the formation of perivascular and hypoxic niches [47,48].

### 2.3. Angiogenesis as a Plausible Target in Glioblastoma Therapy

The dependence of tumor growth and metastasis on angiogenesis, which has been thoroughly demonstrated in murine models, has provided an important rationale to a new kind of therapeutical approach in different kinds of cancer. Even in brain tumors the strategy of targeting blood vessels has always been full of attractions; the anti-angiogenic therapy rationale in malignant brain tumor is based on the following principles: (i) the high vascularity found in malignant gliomas; (ii) the possibility of avoiding the issues related to the passage through the BBB, as opposed to many chemotherapy agents; and (iii) the normalization of the vascular network, which leads to a synergistic effect with other therapeutic agents, when applied together. Moreover, the anti-angiogenic therapy can represent an indirect way of targeting GSCs, because of their involvement in glioblastoma resistance to radio- and chemotherapy [49]. Given this perspective, two classes of drugs have been approved for the treatment of cancers: the monoclonal antibody Bevacizumab (Avastin^®^, Roche), which targets and neutralizes VEGF, and VEGF-linked tyrosine kinase inhibitors (TKIs), including Sorafenib (Nexavar^®^, Bayer-Onyx Pharmaceuticals), Cediranib (Recentin, AstraZeneca) and Sunitinib (Sutent^®^, Pfizer). [43] While Bevacizumab is usually given in combination with other drugs (such as Irinotecan, Etoposide, Temozolomide or Fotemustine) to increase its efficacy, with a toxicity that is acceptable, TKIs as monotherapy show their effect both on neoplastic and stromal cells [50,51]. The main mechanism by which these drugs act on glioblastoma has been thoroughly studied and characterized as vascular normalization: it consists of a focalized effect on newborn vessels, while leaving mature vessels unaltered [52]. Therefore, as observed by Batchelor et al. and fully described by Jain et al., vascular normalization leads to an increase in tumor perfusion and oxygenation, which breaks the vicious circle started by hypoxia [53,54]. Some researchers argue that normalization followed by chemo- or radiotherapy should be the main target of any anti-angiogenic treatment, even for therapies with target other than VEGF. As a matter of fact, when combined, these drug regimens lead to GEC sensibilization to cytotoxic treatment, particularly in non-metastatic brain tumors; moreover, following radiotherapy, anti-VEGF treatment causes a significant decrease in the expression of VEGF in glioblastoma cells [43]. Finally, an important speculation around these drugs is that they could lead to the disintegration of the perivascular niche, resulting in one of GSC ideal habitat loss and, as a consequence, their eradication [55]. While acknowledging this, it is vital to keep in mind the paradox linked with these drugs: they are designed with the aim of disrupting the vascularization while, at the same time, they need it to reach the site to perform their effects. The only way to solve this apparent problem lies in their judicious use, at the correct dose and in the correct therapeutic range, with the aim of avoiding their side effects, as demonstrated in several preclinical studies on murine models with breast cancer or glioblastoma cellular lines [56].

However, anti-angiogenic therapies have not led to a significant improvement in overall survival (OS) in glioblastoma patient, both newly diagnosed and relapsed. In 2018 Ameratunga et al. released a meta-analysis comparing 11 multi-center and/or international studies, with the aim of acknowledging whether a difference could be found in terms of OS and PFS between glioblastoma affected patients treated with the combination of anti-angiogenic therapy and gold standard regimen compared to the standard therapy alone. The authors concluded that various anti-angiogenic drugs did not show a significant increase in OS, while it is also evident that they increased PFS. This is presumably related to both the ability of the tumor to escape the effects of therapy and to the side effects of therapy on vascularization. The problem arises from glioblastoma localization and activity: above all, these drugs can give important side effects such as intracerebral hemorrhage, arterial thromboembolic events or, less frequently, posterior leukoencephalopathy syndrome (RPLS), that can present with headache, seizures, lethargy, confusion, blindness and other visual and neurological disturbances [44,57]. On the other hand, the ability of glioblastoma of evading therapies effect is well known. Notably, the use of anti-VEGF drugs, both in preclinical and in clinical trials, seems to select more aggressive neoplastic clones, with a more exacerbated invasiveness phenotype [58,59]. This confirms what has been previously reported: targeting angiogenesis could theoretically be a good way to attack glioblastoma. However, the implied drugs should also influence GSCs, otherwise it will at least be difficult to overcome glioblastoma resistance to therapy. As a result, further studies should be undertaken to fully comprehend the eventual clinical importance of these drugs in glioblastoma therapy.

## 3. Metformin

The first evidence of metformin as a potential therapeutic treatment dates back to 1922, when the chemists Emile Werner and James Bell observed its ability in reducing glucose concentration in rabbits, without affecting heart rate and blood pressure. Afterwards, it was introduced as a medication in France in 1957 and the United States in 1995 [15]. The main properties of metformin are listed in Table 1.

Metformin is a biguanide originating from Galega officinalis, used in folk medicine for several centuries, and is currently used for the treatment of type 2 diabetes mellitus (T2DM) [60,61,62]. The action of metformin determines a decrease of fasting and post-fasting glucose, glycated hemoglobin (HbA1c, 1–1.5%), considered as an additional marker of diabetes, and insulin resistance [15]. In addition, metformin proved also to reduce glycogenesis, by increasing glucose uptake into muscle cells and leading to a decrease in blood glucose and insulin level, thanks to activation of adenosine monophosphate-activated protein kinase (AMPK). Of relevance, it has been shown that metformin exerts beneficial effect also on hyperlipidemia and non-alcoholic fatty liver disease (NAFLD) [63,64], and is currently prescribed to patients suffering from polycystic ovarian syndrome (PCOS) [65]. The main difference between metformin and the other anti-diabetic compounds refers to its minimal side effects and its low cost. Further, there is evidence of increased survival of patients assuming metformin [66]. Recent epidemiologic studies confirmed that the administration of metformin to diabetic patients at the standard clinical dose (1500–2250 mg/day), succeeded in reducing cancer incidence and/or related mortality. Experimental data also confirmed the activity on metformin in arresting cancer progression, including pancreatic, prostatic, gastric, breast and uterine cancer, both alone and in combination with radiotherapy [67,68]. Notably some of these studies present some methodological limitations, as most have been conducted retrospectively with samples registered from hospital rather than from population, potentially introducing selection biases. Some studies did not exclude patients with previous diagnosis of cancer, which represent subjects with potential for recurrence. Other studies analyzed subjects exposed to different treatments for diabetes, which render the association of metformin quite doubtful. However, there is supportive evidence that metformin could be a potential add-on drug in cancer therapy as it may prevent multidrug resistance, block NAD+ regeneration that leads cell death and improves radiotherapy cell sensibility. Additionally, metformin causes ROS formation, a toxic cell agent that increase DNA damage in cancer cells [69].

### 3.1. Molecular Mechanism of Metformin Effect

**Figure 2:**
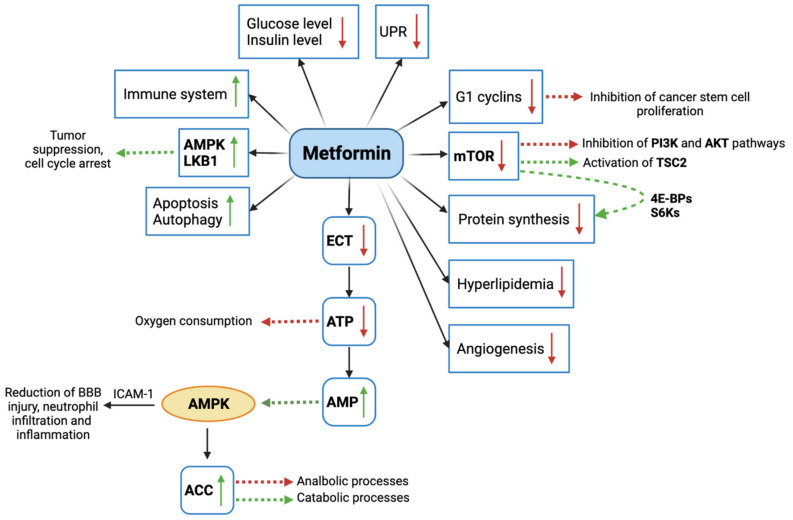
Graphic representation of molecular mechanisms mediated by metformin. mTOR: mammalian target of rapamycin; PI3K: phosphoinositide 3-kinase; AKT: Protein kinase B; TSC2: TSC Complex Subunit 2; 4E-BPs: 4E-binding proteins; S6Ks: ribosomal protein S6 kinase; ECT: electron transfer chain; ATP: adenosine triphosphate; AMP: adenosine monophosphate; AMPK: adenosine monophosphate-activated protein kinase; ACC: acetyl coenzyme A carboxylase; ICAM-1: intercellular adhesion molecule-1; LKB1: Liver Kinase B1; UPR: unfolded protein response. Green arrows indicate activation and/or increased expression, red arrows indicate inhibition and/or decreased expression.

The transport of metformin is managed by two types of transporters: on the luminal side of the enterocytes, the uptake is mainly mediated by the plasma membrane monoamine transporter (PMAT), whereas in other compartments, including the basolateral and luminal side of the enterocytes, the superfamily of transporters exploited by metformin is those of organic cation transporters (OCT), of which Oct1 and Oct3 are the most important, located in the muscle, heart, kidney and liver cells [70]. Metformin continues its pathway into the liver where its uptake is due to Oct1/3 and the extrusion using the transporter Multidrug and Toxin Extrusion 1 Transporter (MATE1). Finally, metformin is excreted via the urinary system, as Oct2 allows metformin intake in the renal epithelial cells, then excreted into the urine by MATE1/2k [71]. As widely reported, the efficacy of metformin as anti-cancer compound is mainly exerted by the following mechanisms, schematically represented in Figure 2: (1) decrease of blood glucose and insulin levels; (2) activation of AMPK and LKB1; (3) inhibition of mTOR signaling; (4) cell cycle arrest; (5) apoptosis and autophagy triggered by p53 and p21; (6) stop of protein synthesis; (7) immune system activation; (8) cancer stem cell destruction; (9) inhibition of unfolded protein response (UPR); (10) diminution of hyperlipidemia; (11) angiogenesis prevention [15].

Primarily, the intracellular introduction of metformin via Oct-1/3 leads to the blockade of the complex I of the electron transfer chain (ECT), with the consequent decrease of oxygen consumption and ATP production, which in turn determines a cellular stress condition [72,73,74]. The reduction of ATP also causes an increase of adenosine monophosphate (AMP), which is able to activate AMPK that, acting as an energy sensor, regulates the amount of energy in the cells [74,75,76]. Another mechanism mediated by metformin is the activation of the serine/threonine kinase LKB1 (Liver Kinase B1), a known tumor suppressor that play an important role in controlling cell cycle, apoptosis and cell autophagy by also regulating AMPK activity. One of the most interesting anti-tumor effects of metformin regards the disruption of intracellular signaling mediated by the mammalian target of rapamycin (mTOR) [77]. Generally, the food uptake determines the increased liver cell expression of insulin-like growth factor (IGF), IGF-receptor and insulin-receptor. This in turn lead to the activation of a signal transduction starting from the insulin receptor substrate (IRS), involving the phosphoinositide 3-kinase (PI3K) and Akt (PKB, protein kinase B) and inactivating the TSC Complex Subunit 2 (TSC2), known as a tumor suppressor. The activation of mTOR as an indirect result of the signal transduction, inhibits TSC2 and promote cell growth and proliferation. Several studies reported that cancer risk and progression is associated with mTOR activation. Therefore, it is plausible that metformin anti-cancer effects are associated with the inhibition of mTOR activity [67]. The effect of metformin on cell growth is also mediated by the reduced expression of G1 cyclins, which alter cell cycle progression [78]. Mechanistically, increasing evidence demonstrated that the anti-cancer activity of metformin can be exerted by an insulin-independent or direct mechanism, and an insulin dependent one. The insulin-independent mechanism depends on AMPK activation and mTOR inhibition, which results in the activation of TSC2 as described above. It has been shown that the inhibition of mTOR lead also to reduction of the 4E-binding proteins (4E-BPs) and the ribosomal protein S6 kinase (S6Ks), responsible for protein synthesis and cell proliferation. In parallel, the activation of AMPK has been shown to decrease the activity of the fatty acid synthase, whose upregulation in tumor leads to the increased production of de novo fatty acids [79]. Another study proved that metformin-induced AMPK increase can activate acetyl coenzyme A carboxylase (ACC), which regulates cellular metabolism by reducing anabolic processes and increasing catabolic ones [79,80]. The indirect mechanism of metformin activity consists of the prevention of the transcription of gene responsible for glycogenesis in liver cells, caused by AMPK activation. As a result, glycogenesis decreases and glucose uptake in muscle cells increases, with a subsequent decrease of blood glucose levels and insulin level increase. Due to the high expression of insulin receptors in cancer cells, the high concentration of insulin in blood determines high mitogenic effects, consisting of cell proliferation and survival.

Among the adverse prognostic factors recognized in cancers as breast, colon and prostate cancer, high insulin levels have been widely described [81]. This evidence prompted the potential use of metformin as a safe drug to lower circulating insulin levels not only in diabetic patients [82], and in turn to counteract cancer progression, as discussed below.

Furthermore, aside the activity of metformin in glucose-related cellular mechanisms, Liu et al. reported that metformin also proved to attenuate BBB disruption in mice with transient middle cerebral artery occlusion, by diminishing neutrophil infiltration, preventing endothelial injury, and consequently improving long-term recovery. These effects, mediated by an AMPK-dependent intercellular adhesion molecule-1 (ICAM-1) may counteract glioblastoma progression, by alleviating the pro-inflammatory microenvironment and protecting the vascular compartment, also involved in angiogenesis [83].

### 3.2. Evidence of Metformin Potential on Gliomas

The intuition of a possible use of metformin as an add-on to chemotherapy in several types of cancers, derived from the observation of the significative preventive and/or beneficial effects on diabetic patients. The disorders of carbohydrate metabolism represent a serious concern in medicine, as they affect a constantly increasing number of patients, so that it has been estimated that by 2030, 454 million adults worldwide will experience diabetes [84]. It is widely recognized that these kinds of diseases, including T2DB and obesity, may contribute to tumor onset, being also reliable factors for poor prognosis in patients with gliomas [81]. A study conducted by Chaichana et al. on 182 patients with low-grade gliomas (WHO grade (ii)) reported that constant hyperglycemia resulted in a decrease of treatment efficacy, with consequent decrease of patient survival and increased tumor recurrence [85]. Analogous results were obtained examining patients with high-grade gliomas and glioblastoma, so that patients with glioblastoma and diabetes showed a worse prognosis [86,87,88]. These observations suggested the potential beneficial effects of drugs lowering blood glucose concentration in the treatment of glioma. Pyaskovskaya demonstrated that the cytotoxic activity of metformin is due to a reduction in glucose levels in the tumor milieu which makes the cells particularly responsive to this drug [89]. Noteworthy, the evidence that anti-cancer treatments used to prevent brain edema, like steroids (e.g., dexamethasone), may impact carbohydrate metabolism, and deserve attention as their use may cause hyperglycemia and in turn may worsen patient prognosis. However, as proposed by Derr et al., the proper control of steroid dose can effectively bypass hyperglycemia adverse effects, improving patient prognosis and clinical outcome [87]. In this contest, Adeberg et al. treated a cohort of 276 patients with glioblastoma and diabetes, observing an increase of PFS when metformin was administered [90]. Similarly, an improved OS and PFS was observed by Seliger et al. in 1093 patients suffering from high-grade gliomas (WHO grade (iii)) and treated with metformin [91]. Unfortunately, a further study by Seliger et al. to confirm these results on 1731 glioblastoma (WHO grade (iv)) patients revealed no significant correlation between OS, PFS and the use of metformin as monotherapy [92], suggesting the need for further studies to examine this discrepancy.

Of relevance, several studies also demonstrated a significant efficacy of metformin in cancer prevention, as diabetic patients treated with metformin for long time present a reduced probability to develop cancer compared to controls, including gliomas [93].

### 3.3. Pre-Clinical Studies on the Efficacy of Metformin on Glioblastoma

The potential effect of metformin in inhibiting tumor cell growth has been described in melanoma, lung, prostate, pancreatic, colon, breast and endometrial cancer [93,94,95,96]. This effect was visible in both in vitro and in vivo experiments, by using metformin alone or along with radiotherapy [15]. Promising observations have been made also for gliomas, in terms of inhibition of tumor cell proliferation, differentiation and invasiveness, and also apoptosis and autophagy [41,58,97,98]. Metformin proved to also increase the effectiveness of standard glioma therapies [99]. As aforementioned, the standard therapy for glioblastoma consists in the surgical resection of the mass, followed by the administration of radiotherapy and chemotherapy with TMZ. However, it is well known that, because of the nature of this kind of tumor, a condition of resistance inevitably occurs, leading to relapse. Moreover, given what has already been mentioned, particularly regarding the metformin effect on apoptosis, it is difficult to argue that the standard strategy could be replaced by only introducing this drug. However, it has been proven that metformin can increase tumor cells sensibility to chemo- and radiotherapy, thus generating interest primarily as an add-on therapy in glioblastoma. Several studies have shown that metformin and TMZ co-administration leads to a synergic response by glioblastoma cells, with an increase in mortality both in sensitive (with hypermethylated MGMT promoter) and in resistant cells to TMZ [61,99,100]. Lo Dico et al. demonstrated in vitro how metformin can reverse resistance to TMZ, even in hypoxia, by modulating the activity of HIF-1α. Furthermore, using two different cell lines, they showed that TMZ and metformin have a marked pro-apoptotic activity and that the addition of the PI3K-inhibitor boosts this activity, affecting both TMZ-responsive and resistant cells [101]. Unfortunately, there are not many observational studies related to the potential importance of metformin therapy in patients with glioblastoma. Instead, given the previously obtained results in other kinds of tumor, the research has started from preclinical studies [16]. The main in vitro effects of Metformin on glioblastoma are summarized in Table 2.

One of the major implications in preclinical studies is dose administration. Typically, significantly higher doses are administered in vitro and in vivo than the amount of metformin used to treat patients with T2DM. In vitro, cells grow in non-permissive conditions. To ensure their survival and expansion, it is necessary to add high doses of glucose, growth factors and hormones. The result of these factors is a decrease in cell responsiveness to administered therapies. Typically, in vitro analyses of tumor cells show an active metformin range of 1–40 mM, compared to 2.8–15 µM in the plasma of T2DM patients [111]. On the other hand, Chandel et al. group in 2016 demonstrated how micromolar plasma concentration of metformin in a mouse model had an antitumoral function. By the administration of 250 mg/kg of metformin in the mouse model, the plasmatic and liver concentrations reached 5 µM and 40 µM respectively, comparable to human concentration [112]. In this regard, Sesen at al. administrated 300 mg/kg of metformin to reduce tumor growth. This group claims that the metformin doses administered in diabetic patients is the minimum required for the glycemic control. Additionally, they argue that metformin treatment in diabetes sufferers is chronic, whereas a higher dose could be administered acutely in GMB sufferers without liver damage [99].

### 3.4. Metformin Effects on GSCs

The definition of GSCs is a dynamic concept. GSCs are defined as those subpopulations of neoplastic cells that share properties with the sane counterpart of stem cells (such as the ability to regenerate and to differentiate into different cell lines) and have the ability of generating neurospheres in vitro or to develop a glioblastoma when transplanted in immunodeficient mice. However, even though several markers (such as CD133 and CD15) are used in in vitro models to recognize them, it is well known that, because of the plasticity of these cells, these markers are not always expressed [113,114,115]. Several studies have evaluated metformin activity on GSCs, both in vitro and in vivo by xeno-transplanting. The rationale of such studies lies in the fact that metformin and TMZ may act synergically, leading to the eradication of chemo resisting glioblastoma cells. The combined treatment has both an AMPK-dependent and independent effect in inhibiting cell growth, by inhibiting mTOR pathway or the whole Akt pathway, on which mTOR is intercalated, respectively. Metformin is the main actor leading to this condition: as a matter of fact, it is well proven that TMZ induces a time-dependent increase of Akt when used in monotherapy, while the use of metformin inhibits it in a time- and concentration-dependent way [116]. To be fair, Wurth et al. previously concluded that metformin could significantly lower Ki67, a cell proliferation marker widely used to characterize glioblastoma, and potentiate TMZ apoptotic activity, through an AMPK mediated mechanism, in a dose- and time-dependent way. In these two studies, Wurth et al. have shown a considerable activity of metformin on GSCs rather than on glioblastoma differentiated cells, opening the street to the following studies aiming to investigate this specific effect [117,118]. On this matter, in 2012 Sato et al. proved that metformin could induce GSCs differentiation through a FOXO3-mediated pathway. FOXO3 is a protein intercalated on AMPK pathway and it is activated by it. The activation of the axis inhibited neurospheres formation and stemness marker BMI1 in vitro and increased differentiation markers like Glial fibrillary acidic protein (GFAP) for astrocytes and β-3-tubuline for neural cells. Depending on metformin dose, also tumor development after the xenotransplantation of GSCs in immunodeficient mice was delayed or blocked. Moreover, systematic administration of metformin led to interesting effects on murine model survival, which increased in a time- and dose-dependent way [108]. Leidgens et al. in 2017 proved that signal transducer and activator of transcription 3 (STAT3) is another important mediator in maintaining GSCs stemness; as a matter of fact, such enzyme acts on the progression of cell cycle, regulating it through the interaction with the adjacent cells. In the study, the authors proved that metformin inhibits this protein through its phosphorylation, causing the loss of stemness features and starting a pro-differentiative and pro-apoptotic process. It was previously proven that the mechanism was a consequence of AMPK activation induced by the drug; however, the authors proved that metformin itself could directly phosphorylate STAT3 on its Y705 binding site (whereas Ser727 was the phosphorylated site after AMPK activation) [109]. Moreover, several recent studies have also proven that metformin suppresses the epithelial mesenchymal transition (EMT), a vital process for neoplastic cells to develop an invasive phenotype. In 2018, Yuan et al. showed that a consequence of this drug administration was the decrease in EMT markers in GSCs, with the suppression of both mRNA and protein levels of Vimentin (an adhesion protein mainly expressed in mesenchymal cells) in favor of E-Cadherin (the epithelial counterpart). The molecular effect was proven to be on the YAP-Hippo axis, a well-known pathway that induces EMT. Indeed, by phosphorylating YAP, the drug prevents it from moving from the cytoplasm to the nucleus and avoids its activity as a transcription modulator in gliomas; thus, it lowers the activity of all the downstream molecules, decreasing its pro-EMT activity. The main prove of this effect was that, increasing the levels of YAP5SA (a downstream YAP target), EMT proceeded even though metformin was being administrated [110]. Finally, in 2014, Gritti et al. designed a study to understand why metformin action is more selective on neoplastic cells and GSCs, leaving other cells undamaged. It was proven that the drug acts on the chloride intracellular channel 1 (CLIC1), which shows a functional expression, meaning that it is expressed only when it must act to allow the transition from G1 to S phase of the cell cycle. In that circumstance, the protein, normally present only in the cytoplasm, translocases to the plasma membrane and starts a chloride current, which is vital to complete the transition. The transient activation of the channel allows metformin to bind the Arg29 domain (on the outer layer of the membrane), stabilizing the close state or obstructing the channel [102]. Analysis on mRNA revealed a correlation between glioblastoma malignancy and expression of CLIC1. In detail, this correlation is present in both human glioblastoma and experimental models [113]. However, because the downstream pathway of the channel is not known yet, it is not clear what is the purpose of these metformin effects and more studies should be conducted to deeper investigate. Evidence of selectively of metformin is demonstrated by its specific action versus GSC cells CD133+ (GSC marker). Metformin treatment demonstrated a reduction of cell growth only in CD133+ compared to CD133- and a lack of proliferation in human stem cell [119].

### 3.5. Could Angiogenesis Be a New Target for Metformin in Glioblastoma Therapy?

**Figure 3:**
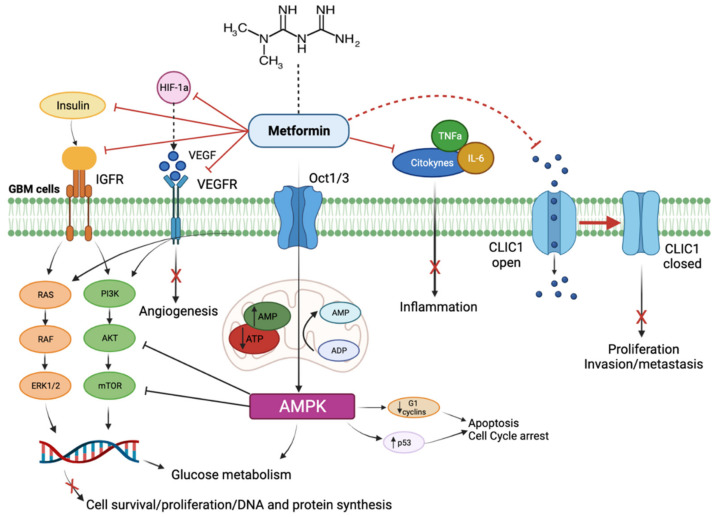
Schematic representation of cellular and molecular effects of metformin on glioblastoma cells. As described in the text, metformin acts by inhibiting IGFR- and VEGFR-mediated pathways, which physiologically lead to angiogenesis, cell proliferation and survival. Furthermore, metformin decreases inflammation and promotes cell cycle arrest and tumor cell apoptosis, by inhibiting cytokine activation and by maintaining the chloride channel 1 (CLIC1) in a closed state.

Metformin could potentially play an important role also in hindering the pathways related to tumor angiogenesis, which is increasingly considered to be a vital process in cancer growth and metastatic ability. As was mentioned before, angiogenesis is significantly linked with the processes of inflammation and hypoxia. Based on the previously known effects on nuclear factor kappa-light-chain-enhancer of activated B cells (NF-kB) and tumor necrosis factor (TNFα) or rather on HIF (which increases VEGF expression), the speculation that metformin could prevent and disrupt angiogenesis is not surprising [120,121]. Starting from this concept, some studies have already tested the effects of metformin in other tumor ECs, with promising results. Xavier et al. in 2010 have evaluated the relationship between inflammation and angiogenesis, by transplanting polyester-polyurethane sponges and analyzing the consequent accumulation of inflammatory cells and the development of vessels through several indicators (hemoglobin, myeloperoxidase, N-acetylglucosaminidase and collagen). In this study, the authors explored the hypothesis that metformin (in doses of 40–400 mg/kg, consistently with those commonly administered in the murine model) could impact neo-angiogenesis, by affecting the expression of pro-angiogenic and pro-inflammatory molecules. A significant decrease in hemoglobin levels and chemokines, such as CCL2 and transforming growth factor beta 1 (TFGβ1) was observed on the sponge, while no effect was noted on VEGF [122]. Subsequently, Dallaglio et al. carried out a study on the effects of metformin on ECs and angiogenesis, with the aim of acknowledging the dose and time-dependent effects. The first observation they made was that the treatment resulted in a decrease of ECs invasiveness and proliferation, by exerting a more cytostatic rather than cytotoxic effect. As a matter of fact, after the administration of the drug at the dose of 1mM on a line of human vascular endothelial umbilical cells (HUVECs), there was a considerable decrease in the levels of both mRNA and protein Cyclin D1 and CDK4 kinase (factors which are commonly involved in the cell cycle). Several time-dependent effects were observed in HUVECs and breast cancer or prostate line co-cultures: in the first 6 h, some genes indicating a pro-angiogenic effect, such as VEGF-A, prostaglandin-endoperoxide synthase 2 (PTGS2), which encodes COX-2, coagulation factor III, thromboplastin (FIII) and ADAM Metallopeptidase with Thrombospondin Type 1 Motif 1 (ADAMTS1), were over-expressed. On the other hand, after 24 h, these levels went back to normal or, rather, were downregulated. Secondly, a decrease of 12 proangiogenic genes expression was observed between 6 and 24 h of treatment, among which ADAMTS1 and VEGF-A were significantly downregulated; moreover, even genes like Fms related receptor tyrosine kinase 1 (FLT1, VEGF receptor 1), WARS (tryptophanyl-tRNA synthetase), protein kinase D1 (PRKD1) and spermidine/spermine N1-acetyltransferase 1 (SAT1), which are all involved in angiogenesis promotion, were significantly reduced. However, the in vitro study showed that metformin had the opposite effect on neoplastic cells and ECs. In fact, in the ECs the drug lowered some pro-angiogenic factors like matrix metallopeptidase 8 (MMP8), while increasing the levels of some others (angiopoietin 1 and 2, IL8, endothelin 1); moreover, it increased the levels of some anti-angiogenic factors (activin A and TIMP metallopeptidase inhibitor 1, TIMP1). In neoplastic cells, the vice versa always appeared to happen. In addition, an important increase in VEGF-C (a pro-angiogenic factor) was demonstrated compared with the ECs. These effects were, at least partially, modulated by AMPK. Finally, the Matrigel pellet in vivo study showed that metformin decreased aberrant neo-angiogenesis: by xeno-transplanting the Matrigel in murine models and by measuring the levels of CD31 (which is a typical ECs marker), it was observed that, at a 2mg/day dose, metformin could lead to a significantly lower level of CD31 positive newborn vessels [123]. On the other hand, a study by Orecchioni et al., in which several co-cultures of breast cancer lines and white adipose tissue were analyzed, demonstrated that metformin acts on neo-angiogenesis and on metastasis by a simultaneous effect both on neoplastic and microenvironment cells (which, in the experiment, were represented by the adipose tissue). By using a proteomic assay, particularly on neoplastic tumoral, the authors analyzed the expression of several angiogenesis-involved genes, such as insulin-like growth factor binding protein 2 (IGFBP2), platelet-derived growth factor (PDGF), VEGF, Angiogenin, MMP9 and endostatin, observing a significant decrease in their levels. Moreover, in accordance with what was observed by Dallaglio, the effects were not associated with an increase in the apoptotic cells fraction: indeed, the expression of several protein levels, such as Serpin E1 or IL8, were not or were slightly decreased after the drug administration. Similar effects were obtained in neoplastic and adipose tissue cells co-culture. Finally, metformin administration significantly lowered microvascular density, with a significant decrease in the CD31 positive component, while the pericytic population was not affected [123,124]. Taken together, these results (schematically illustrated in Figure 3) hold hope for a major anti-angiogenic effect of metformin: however, the paradoxical effect of the drug on neoplastic cells when compared with ECs observed by Dallaglio et al., in agreement with several other studies in the literature, remains an unresolved question and demands further investigation to achieve certainty on this effect [123].

### 3.6. Clinical Trial with Metformin in Glioblastoma

To date there are some studies for clinical trial of metformin in glioblastoma. Most of the cancer clinical trials of metformin use the same doses typically used to treat diabetes. Conducted by Chen K et al., there is a phase 1 led-in phase 2 study where metformin, TMZ, memantine and mefloquine are administrated to glioblastoma patients. These studies demonstrated how drugs combo is tolerated compared to traditional treatment. Indeed, there is a phase 1–2 trials to test metformin in glioblastoma-solid tumor patients with IDH1 or IDH2 mutated [125]. The tolerability of this treatment was analyzed in a clinical trial phase 1–2 by Maraka et al. in 2019 on a cohort of 90 patients affect by glioblastoma (NCT01430351) [126]. A retrospective study performed by Salinger at al. in 2019 showed that metformin use yielded favorable results in both tumor survival and progression in subjects with grade III glioma (WHO scale); no statistically significant data on both survival and progression were found for patients with WHO grade IV glioma [91]. In a more recent study, Salinger evaluated the metformin-survival association in a cohort of subjects with newly diagnosed glioblastoma. The results showed that metformin, whether administered alone or in combination with other drugs, did not increase patient survival. Further analysis could be performed to investigate the possible use of this drug in combination with certain particularly responsive types of glioblastoma [92]. A recent phase II clinical trial by the Weill Medical College of Cornell University is recruiting glioblastoma patients with the aim of evaluating the tolerability and the effects of a ketogenic diet in conjunction with metformin (NCT04691960). Another interesting and very recent multicentric phase II clinical trial conducted by the Hospital Foch and the National Cancer Institute in France and named OPTIMUM involves 640 participants with *IDH*-wildtype glioblastoma. Based on the overexpression of mitochondrial markers in IDH-wt glioblastomas undergoing oxidative stress, the study aims to evaluate the effect of metformin as an oral inhibitor of mitochondrial complex I, in combination with radiation and TMZ. The estimated start date is December 2021 and the outcomes regard the assessment of PFS, OS and Overall Response rate (ORR) that will be measured by the RANO (Response Assessment in Neuro Oncology) criteria (NCT04945148). Of relevance, a recent clinical trial assessed the efficacy of metformin as neo-adjuvant compound together with TMZ and hypofractionated accelerated radio-therapy (HART) in 33 patients with glioblastoma. The study confirmed no adverse effects after the use of metformin, confirming its safety and tolerability and validating previous results on favorable outcomes of glioblastoma patients, particularly those with low methylation levels of MGMT (NCT02780024) [127].

## 4. Conclusions

During the last 20 years, several studies have proven that metformin has a wide-ranging antitumoral effect. The repurposing of this type of drug initiated in recent years is showing promising results in the battle against several cancers, with a wide range of molecular effects that could allow metformin to be applied as an effective add-on therapy to the standard of care for many neoplastic lesions. Particularly, in glioblastoma, metformin could strongly help the standard strategy of care to move forward, towards an improvement of the OS and PFS. However, a deeper knowledge of the antitumoral effects of this drug is required, particularly evaluating its ability in inhibiting or damaging neo-angiogenesis. Indeed, because of all the effects metformin has on GSCs and on glioblastoma generally, an eventual anti-angiogenic effect could make this drug even more suitable in the therapy of this kind of lesion. Therefore, we suggest, based on the previous published results on other tumors, to deepen the knowledge on the anti-angiogenic effect of metformin.

## Figures and Tables

**Table 1:** Pharmacokinetics, pharmacodynamics and chemical properties of metformin.

Metformin Properties	
Structure	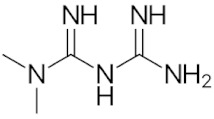
Chemical formula	C_4_H_11_N_5_
Weight	129.16 KDa
Indication	Tablet, oral administration
Associated conditions	T2DM; PCOS
Route of elimination	Kidney
Half-life	Plasma and blood: ~6.2 h Elimination half-life: ~17.6 h
Clearance	510 ± 120 mL/min
Pharmacokinetics	Trough steady-state metformin plasma concentration: 54–4133 ng/ml
Intestinal/hepatic uptake	PMAT, Oct1/3
Carrier	Oct1/3 for absorption; Oct2 for excretion
BBB permeability	Yes

**Table 2:** Overview of in vitro and in vivo studies reporting an anti-glioblastoma effect of metformin.

Metformin Effects	Molecular Pathways	Reference
Metformin specifically acts on neoplastic or glioma stem cells, while not affecting normal cells	Metformin acts by blocking the chloride channel CLIC1. The downstream cascade is yet to be studied	[102]
Metformin alters cells metabolism by acting on ETC I and, consequently, by impairing the ATP/AMP ratio and activating AMPK	Metformin decreases oxidative phosphorylation while increasing the amount of ATP produced through anaerobic glycolysis	[99]
	Metformin decreases the protein synthesis through the inhibition of mTOR while inducing the predominance of catabolic processes	[103]
Metformin increases oxidative stress in glioblastoma cells	Metformin blocks ETC I, generating an impaired mitochondria action and leading to an increase in ROS production	[99]
	Metformin inhibits mitochondrial superoxide dismutase, increasing ROS production	[104]
Metformin inhibits cell proliferation	By activating AMPK, through the phosphorylation of PIKE-A, Metformin inhibits the Akt/mTOR axis	[104]
	By activating TSC2 and RAPTOR, Metformin inhibits mTOR	[105]
Metformin inhibits cell motility and invasiveness	By activating AMPK, through the phosphorylation of PIKE-A, Metformin inhibits the Akt/mTOR axis	[106]
Metformin moderately increases apoptosis	Metformin increases the levels of caspase 3	[86,106]
	Metformin increases the levels of caspase 9	[107]
	Metformin increases the levels of Bax, while reducing the levels of Bcl-2	[99,106]
Metformin increases sensitivity to chemo- and radiotherapy	Metformin inhibits HIF and its downstream effects	[60,101]
Metformin acts on GSCs	Together with TMZ, Metformin inhibits proliferation and promotes apoptosis	[101,107]
	Metformin induces GSCs differentiation by activating FOXO3	[108]
	Metformin induces GSCs differentiation by inhibiting STAT3, through AMPK (phosphorylation site Ser727) or directly (phosphorylation site Y705)	[109]
	Metformin inhibits GSCs EMT through the inhibition of the axis YAP/Hippo	[110]
